# Two Ways to Examine Differential Constitutive Equations: Initiated on Steady or Initiated on Unsteady (LAOS) Shear Characteristics

**DOI:** 10.3390/polym9060205

**Published:** 2017-06-03

**Authors:** Jana Zelenkova, Radek Pivokonsky, Petr Filip

**Affiliations:** Institute of Hydrodynamics, Academy of Sciences of Czech Republic, Prague 16000, Czech Republic; zelenkova@ih.cas.cz (J.Z.); filip@ih.cas.cz (P.F.)

**Keywords:** LAOS, Fourier-Transform rheology, exponential Phan–Tien and Tanner (PTT) model, Giesekus model, Leonov model, modified extended Pom–Pom (mXPP) model, poly(ethylene oxide)

## Abstract

The exponential Phan–Tien and Tanner (PTT), Giesekus, Leonov, and modified extended Pom–Pom (mXPP) differential constitutive models are evaluated in two ways: with regard to steady shear characteristics and with regard to large amplitude oscillatory shear characteristics of a solution of poly(ethylene oxide) in dimethyl sulfoxide. Efficiency of the models with nonlinear parameters optimized with respect to steady shear measurements is evaluated by their ability to describe large amplitude oscillatory shear (LAOS) characteristics. The reciprocal problem is also analyzed: The nonlinear parameters are optimized with respect to the LAOS measurements, and the models are confronted with the steady shear characteristics. In this case, optimization is based on the LAOS measurements and equal emphasis is placed on both real and imaginary parts of the stress amplitude. The results show that the chosen models are not adequately able to fit the LAOS characteristics if the optimization of nonlinear parameters is based on steady shear measurements. It follows that the optimization of nonlinear parameters is much more responsible if it is carried out with respect to the LAOS data. In this case, when the optimized parameters are used for a description of steady shear characteristics, efficiency of the individual models as documented differs.

## 1. Introduction

Differential constitutive models are quite often confronted only with the rheological characteristics of polymeric materials exposed to relatively moderate deformation. However, in polymer processing, we encounter deformation in higher orders. In this respect, a shift from analysis of flow behavior in the purely linear viscoelastic region to the non-linear region was enabled by the onset of more ingenious measurements represented by large amplitude oscillatory shear (LAOS) data (initiated already a few decades ago [1,2,3,4,5,6]). This provides a possibility of more thorough evaluation of the individual constitutive models due to the remarkable extension of applied deformation.

The input harmonic signals for this technique in comparison with the small amplitude oscillatory shear (SAOS) approach exhibit higher intensity in both frequency and amplitude of deformation. It is no longer possible to expect a response in the same functional form as in the case of SAOS measurements. Therefore, more sophisticated methods are required for data processing. The methods introduced in [7,8] enable more developed processing of the “classical” Lissajous–Bowditch plots (relating raw stress signal to strain or strain rate resulting in elastic or viscous Lissajous–Bowditch plots, respectively). These plots document non-linearities by deviation of their closed shapes from the ellipsoidal ones. Further, more sophisticated methods and an application of the new techniques (Fourier-Transform Rheology [9,10,11], stress decomposition [12], Ewoldt quantities [13]) are summarized, e.g., by Hyun et al. [14].

The suitability of modeling the rheological data within a non-linear viscoelastic region (covered by the LAOS measurements) for individual empirical [7,15] and differential constitutive equations [15,16,17,18,19,20,21,22,23,24,25,26,27] has recently attracted attention. Naturally, these analyses are subject to used polymeric materials and to applied forces to which material is exposed (characterized by strain and frequency). Recently, for the case of thixotropic suspensions, Armstrong et al. [28] and Kim et al. [29] tested the efficiency of the selected elastoviscoplastic models (practically comparable within traditional thixotropic measurements) against LAOS measurements and showed significant disproportions in their abilities to describe material structure in more detail.

The aim of this contribution is to evaluate an efficiency of four differential constitutive models (exponential Phan–Tien and Tanner (PTT) [30], Giesekus [31], Leonov [32], and modified extended Pom–Pom (mXPP) [33]) for the prediction of steady and transient rheological characteristics within a non-linear viscoelastic region described by the LAOS data. The material used is a 5 wt % solution of poly(ethylene oxide) in dimethyl sulfoxide and the applied technique is represented by the Fourier-Transform rheology. The emphasis is equally placed both on the normalized stress magnitudes and on the phases of the individual harmonics generated by the original raw time–stress signal. The optimized values of nonlinear parameters of the studied constitutive models are also used for a comparison with the steady shear characteristics and vice versa, the parameters optimized for a description of steady shear characteristics are applied for a comparison with the LAOS measurements.

## 2. Differential Constitutive Models

The extra stress tensor **τ** is calculated as a sum of all contributions from each relaxation elements spectrum

(1) $$\tau =\overset{N}{\underset{i=0}{\sum }}\tau_{i}.$$

The individual extra stress contributions **τ***_i_* are supposed for each mode *i* to fulfil the following relation

(2) τ=G(c−I)

where *G* represents the elastic shear modulus, ***c*** is the symmetrical conformation tensor, and ***I*** denotes the unit tensor. The evolution equation of the differential models can be written in the form

(3) $$\frac{dc}{dt}-\nabla v\cdot c-c\cdot \nabla v^{T}-\frac{\xi }{2}\left(\dot{\gamma }\cdot c+c\cdot \dot{\gamma }\right)+\frac{1}{\lambda }H\left(c\right)=0$$

where ***v*** is the velocity, $\dot{\gamma }$ is the rate of deformation tensor ($\nabla v+\nabla v^{T}$), λ is the relaxation time, and ***H***(***c***) is the dissipative term depending on conformation tensor ***c*** (see Table 1 for the individual models). The parameter *ξ* denotes the non-affine motion parameter (0 ≤ ξ ≤ 2). Its limiting values ξ = 0 and ξ = 2 represent the contravariant (upper convected derivative) covariant (lower convected derivative) form, respectively. The Giesekus, Leonov, and modified XPP constitutive models used in this work suppose ξ = 0 in contrast to the exponential Phan–Tien and Tanner model.

## 3. Material Preparation and Its Rheological Characterization

For experimental measurements, 5 wt % solution of polyethylene oxide (*M*_v_ = 1000 kg/mol, Sigma Aldrich, St. Louis, MO, USA) with dimethyl sulfoxide (DMSO, Penta s.r.o., Prague, Czech Republic) was used. The samples were stirred in a magnetic stirrer (Heidolph MR Hei-Tec, Schwabach, Germany) with the help of a Teflon-coated magetic stick. The stirrer was applied for 24 hrs with a mixing rate of 250 rpm at 60 °C.

**Figure 1:**
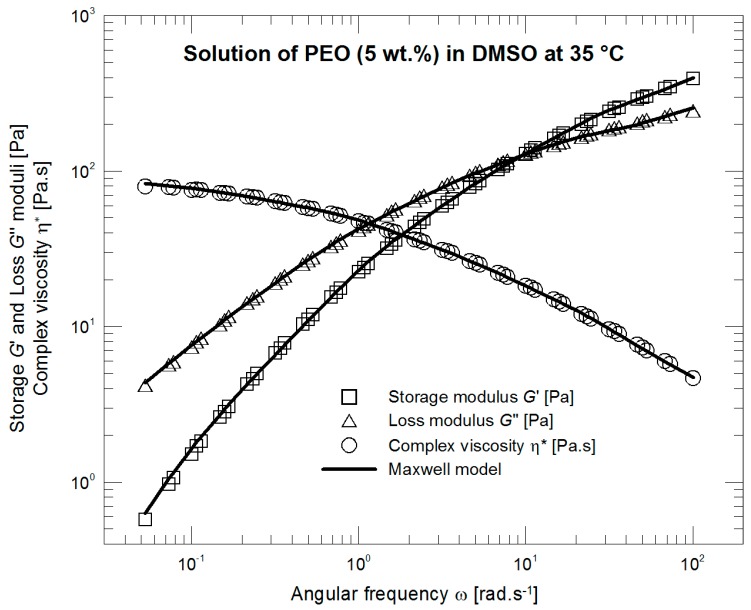
Master curve fitted with the Maxwell model.

**Figure 2:**
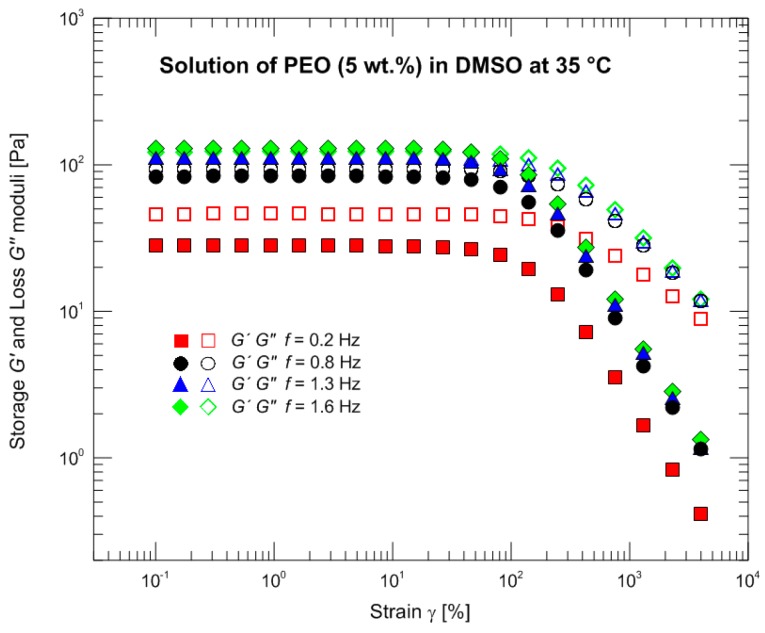
Oscillatory strain sweep tests at four different frequencies.

**Figure 3:**
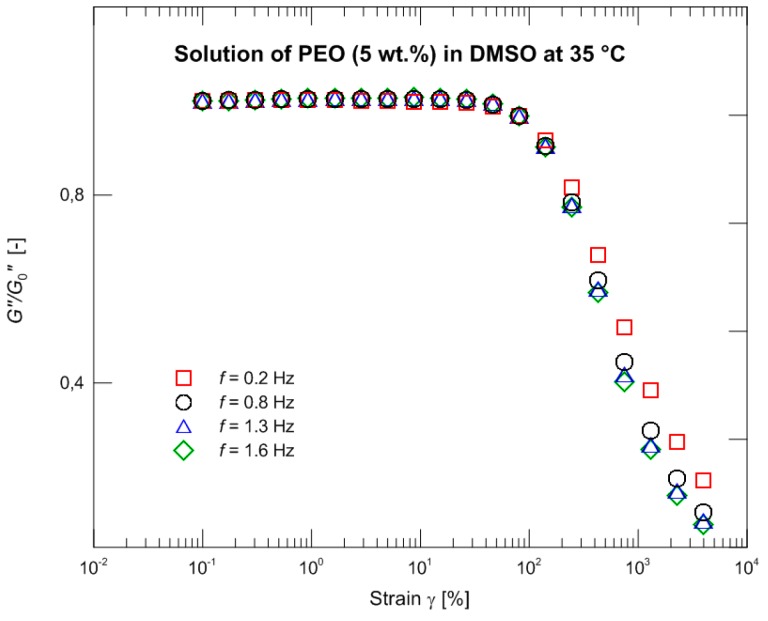
Normalized loss modulus as a function of strain.

The measurements wre carried out with a rotational rheometer MCR Physica 501 (Anton Paar, Graz, Austria) using the Peltier system equipped with the cone-plate arrangement (diameter 50 mm/1°). The outer part (between the edges of cone and plate) of a measured sample in contact with surrounding air was coated by a thin layer of silicone oil (shear viscosity 15 mPa·s) to suppress sample evaporation. The small amplitude oscillatory shear characteristics describing behavior in a linear viscoelastic region were measured at three different temperatures 35, 45, and 55 °C. The master curve was calculated for 35 °C and the linear parameters λ*_i_* and *G_i_* of the Maxwell relaxation spectrum were determined (Table 2, Figure 1). Figure 2 illustrates oscillatory strain sweep tests at four different frequencies: 0.2 (used in the following experiments), 0.8, 1.3 (crossover frequency), and 1.6 (storage modulus exceeding loss modulus). Figure 3 depicting normalized loss modulus as a function of strain documents no presence of strain overshoot phenomenon (in contrast e.g., to xanthan gum measurements [34]) and hence no extra resistance against deformation at the onset of the nonlinear viscoelastic response. Based on these findings, the large amplitude oscillatory shear properties were consecutively measured at 35 °C with a frequency of 0.2 Hz and strains of 50%, 100%, 500%, 1000%, 2000%, and 4000%. The acquired time domain signal was trimmed into whole periods to properly calculate the Fourier transformation. Data processing (data trimming, Fourier transformation, fitting of the individual constitutive models, etc.) was carried out with the help of the MATLAB software (The MathWorks, Inc., Torrance, CA, USA).

## 4. Results and Discussion

Two independent measurements were carried out using 5 wt % PEO in DMSO. First, a “classical” measurement of steady shear viscosity over three decades of shear rate was carried out. Second, the polymer solution was exposed to large amplitude oscillatory deformation under strain γ consecutively attaining values 50%, 100%, 500%, 1000%, 2000%, and 4000% and under frequency *f* = 0.2 Hz. An acquired signal after trimming into whole periods was processed by the Fourier transformation. No indication of non-zero even harmonics causing the unacceptability of measurements [35,36,37] appeared; on the contrary, it was possible to determine sufficiently high numbers of odd harmonics with negligible noise participation.

Efficiency of the exponential Phan–Tien and Tanner (PTT), Giesekus, Leonov, and modified extended Pom–Pom (mXPP) models was tested in two ways. The nonlinear parameters of the models were optimized with respect to a description of steady shear viscosity (SSV); consequently, the behavior of these models with such determined parameters was compared with the data obtained by the LAOS procedure. The other way was arranged in the reverse mode. The nonlinear parameters were determined through the LAOS measurements, and such models were confronted with the measured data representing steady shear viscosity.

### 4.1. Steady Shear Viscosity as Primary Measurement

**Figure 4:**
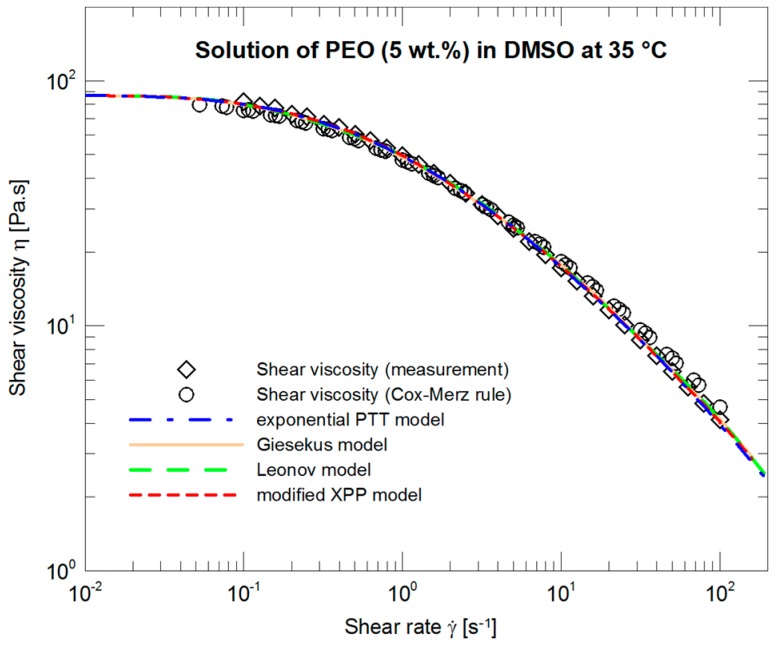
Optimization of the exponential PTT, Giesekus, Leonov, and modified XPP models with respect to the measured steady shear viscosity data.

The courses of the four models, the parameters of which (Table 2, SSV) were optimized with respect to steady shear viscosity measurements, are depicted in Figure 4. All four curves are more or less identical and in very good agreement with the experimental data not only in what concerns the absolute values but also in copying concavity of the experimental data.

**Figure 5:**
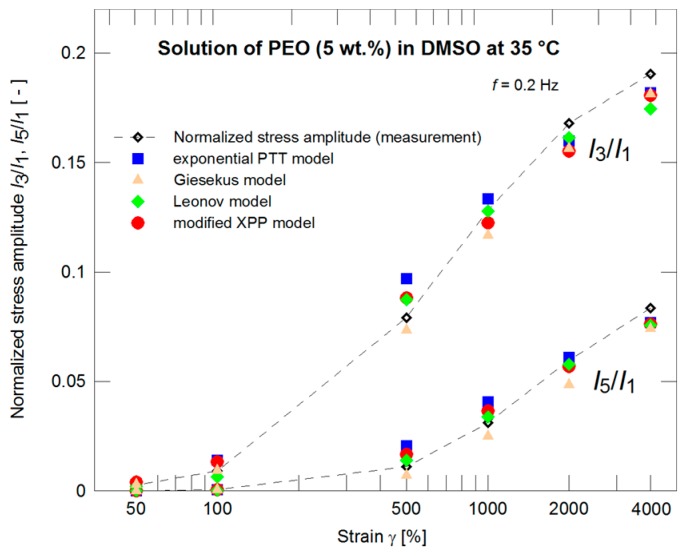
A description of the normalized stress amplitudes by the exponential PTT, Giesekus, Leonov, and modified XPP models using the nonlinear parameters optimized with respect to the steady shear viscosity measurements (Figure 4) for strains of 50%, 100%, 500%, 1000%, 2000%, and 4000%.

**Figure 6:**
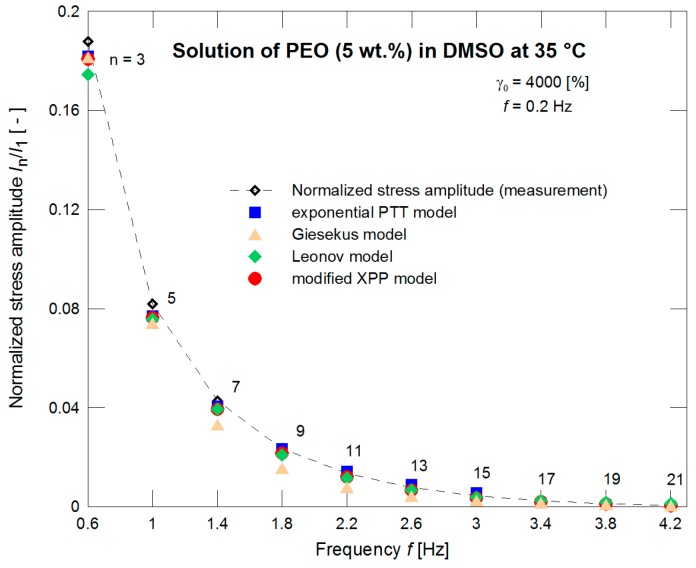
A description of the normalized stress amplitudes by the exponential PTT, Giesekus, Leonov, and modified XPP models using the nonlinear parameters optimized with respect to the steady shear viscosity measurements (Figure 4) for strain of 4000%.

The constitutive models with such pre-determined nonlinear parameters were further used for evaluation of the normalized stress amplitudes *I*_3_/*I*_1_ and *I*_5_/*I*_1_ experimentally obtained through the LAOS measurements for different strains. Behavior of all models seems to be comparable (see Figure 5). If the measurements for strain γ_0_ = 4000% only are taken into account (Figure 6) with the initial 21 normalized stress amplitudes (i.e., initial 10 odd normalized stress amplitudes) then the exponential PTT and modified XPP models have better correspondence to the experimental data.

**Figure 7:**
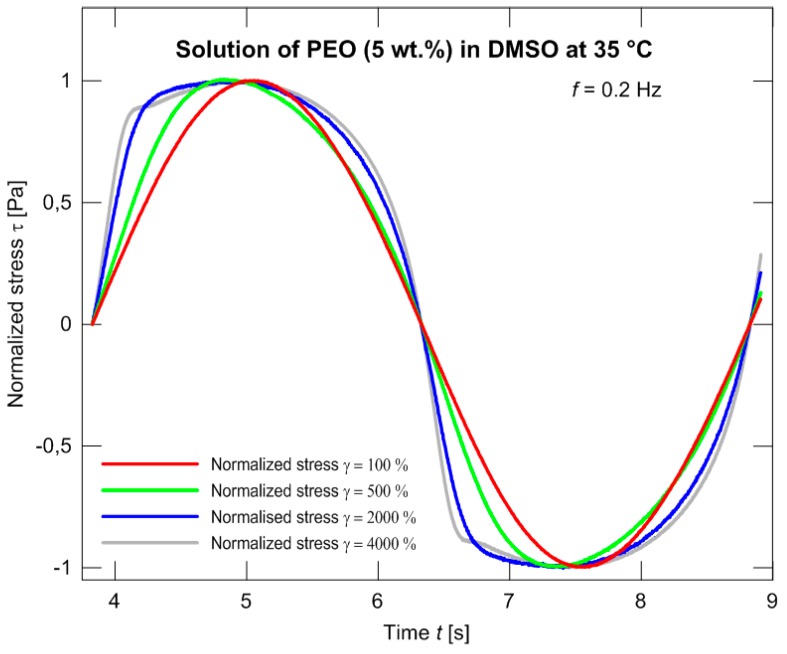
An evolution of the normalized stress curve with increasing strain.

**Figure 8:**
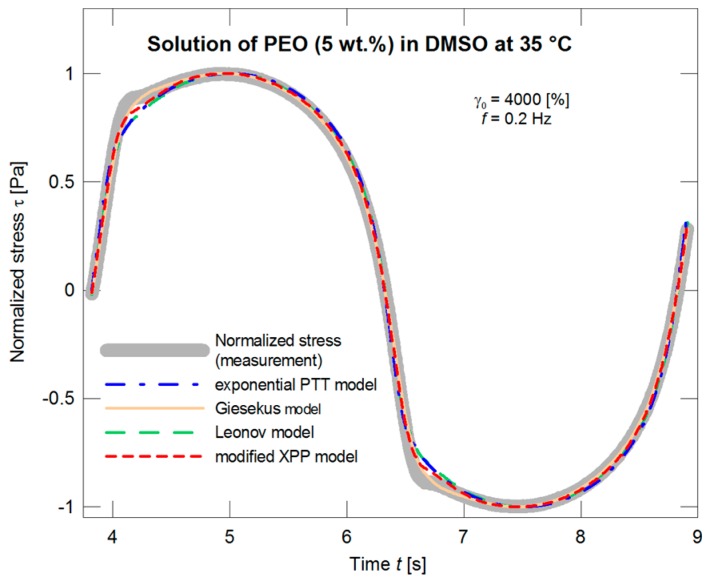
A description of normalized stress by the exponential PTT, Giesekus, Leonov, and modified XPP models using the nonlinear parameters optimized with respect to the steady shear viscosity measurements (Figure 4).

**Figure 9:**
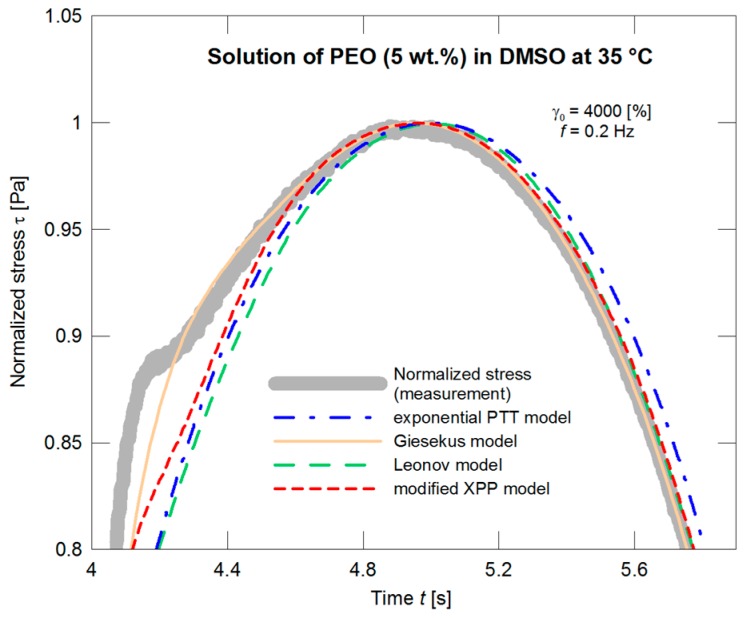
A description of normalized stress by the exponential PTT, Giesekus, Leonov, and modified XPP models using the nonlinear parameters optimized with respect to the steady shear viscosity measurements (Figure 4), the detail with the “protrusion” region.

A substantially worse situation appears if the models are applied in dependence on time. In this case, with the material used, the normalized stress exhibits a “protrusion” (its evolution illustrated in Figure 7), and neither model is able to depict this phenomenon as illustrated in Figure 8 and in more detail in Figure 9.

### 4.2. LAOS Approach as Primary Measurement

**Figure 10:**
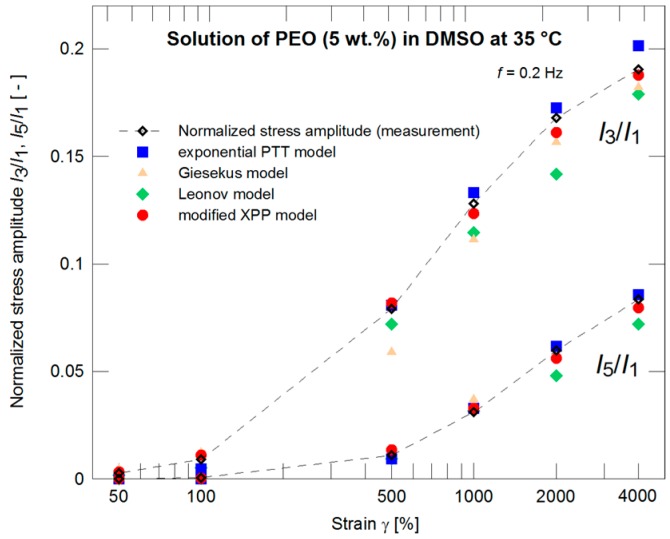
Optimization of the normalized stress amplitudes *I*_3_/*I*_1_ and *I*_5_/*I*_1_ by the exponential PTT, Giesekus, Leonov, and modified XPP models for strains of 50%, 100%, 500%, 1000%, 2000%, and 4000%.

**Figure 11:**
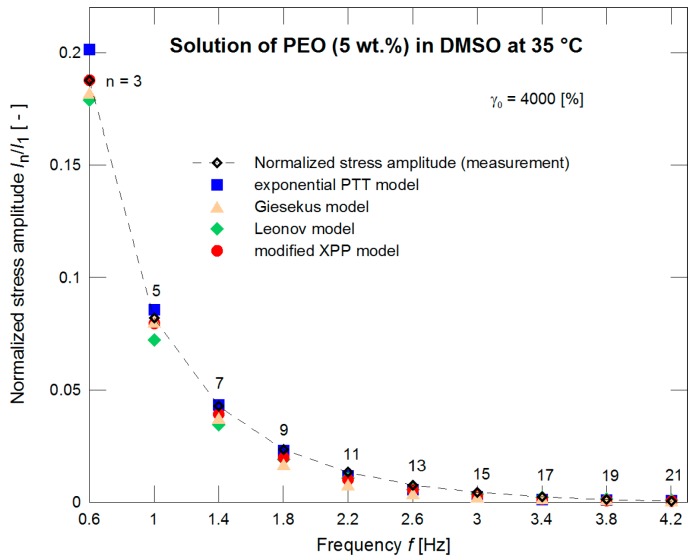
Optimization of the normalized stress amplitudes by the exponential PTT, Giesekus, Leonov, and modified XPP models for a strain of 4000%.

The same material was used in Pivokonsky et al., where it is documented that there is a necessity of using higher harmonics (in the case of the PEO solution, the first 15 harmonics, i.e. the first 8 odd harmonics) for a correct determination of the nonlinear model parameters with simultaneous attention to the phase of the individual harmonics. The nonlinear model parameters were determined for strain γ_0_ = 4000% and frequency *f* = 0.2 Hz. Due to relative simplicity, the other LAOS characteristics at lower strains were neglected during the fitting procedure (determination of nonlinear parameters). The parameters are summarized in Table 2 in the columns denoted as LAOS. A difference in the prediction of normalized stress amplitudes *I*_3_/*I*_1_ and *I*_5_/*I*_1_ (providing no information on phases) for the individual models is depicted in Figure 10. If the nonlinear parameters of the models are optimized with respect to both magnitudes and phases to fit properly the LAOS data, the prediction of the normalized stress magnitudes for γ_0_ = 4000% is shown in Figure 11.

**Figure 12:**
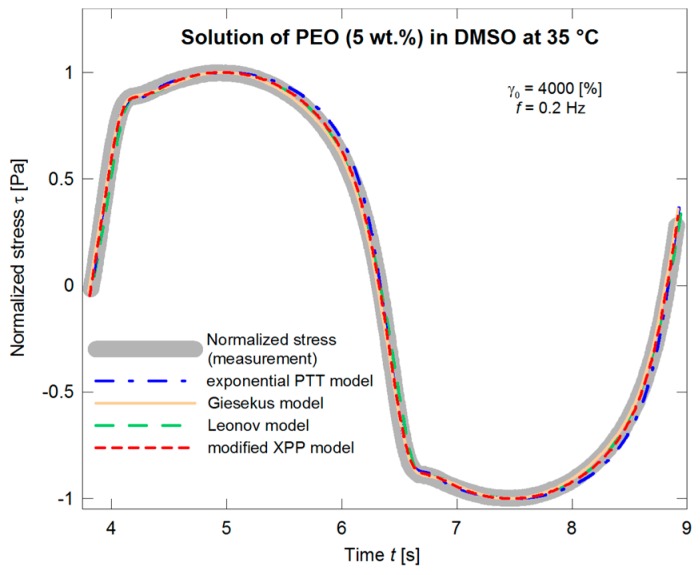
Optimization of the exponential PTT, Giesekus, Leonov, and modified XPP models with respect to the normalized stress amplitude.

**Figure 13:**
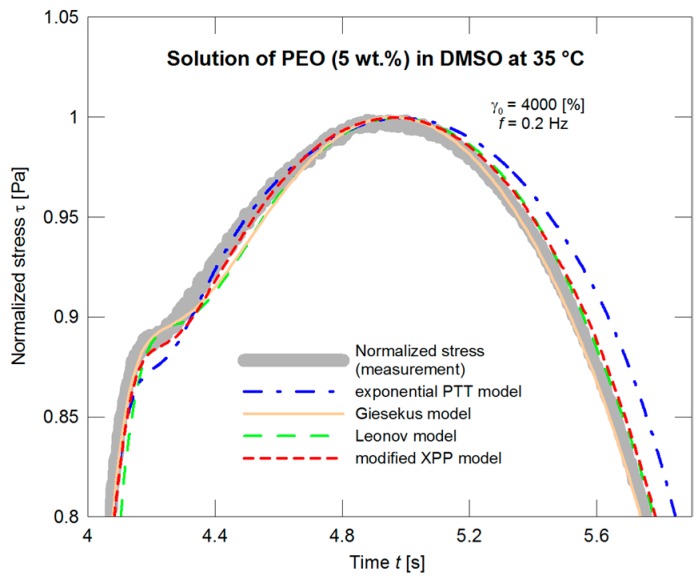
Optimization of the exponential PTT, Giesekus, Leonov, and modified XPP models with respect to the normalized stress amplitude, the detail with the “protrusion” region.

As expected, in this case, the predictions of the models are in much better coincidence with the normalized stress data in dependence on time (Figure 12). Even if the situation is illustrated in more detail (Figure 13), the deviations of the individual models are comparable with inaccuracy of the LAOS measurements.

**Figure 14:**
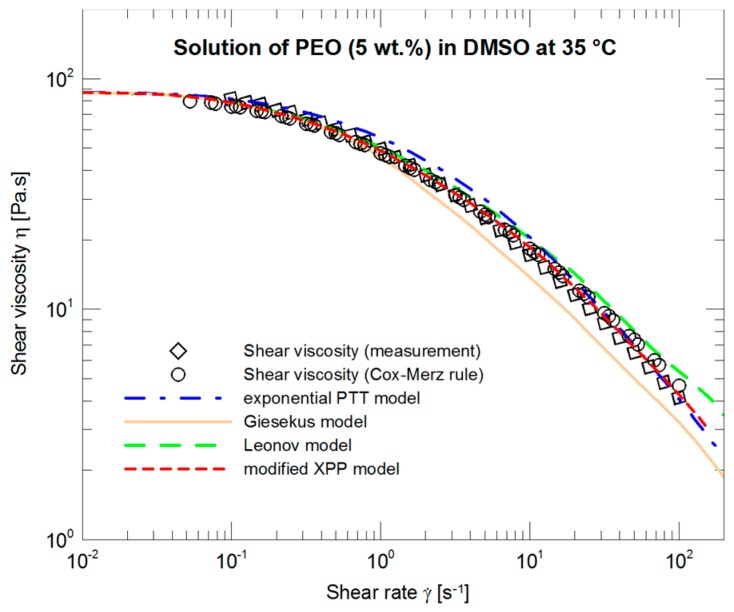
A description of steady shear viscosity by the exponential PTT, Giesekus, Leonov, and modified XPP models using the nonlinear parameters optimized with respect to the LAOS measurements (Figure 11).

The individual models are more diversified if the LAOS-based parameters are applied to a description of steady shear viscosity (Figure 14). The models either over-predict or under-predict the experimental data, and the only exception is represented by the modified XPP model, which not only follows the data but also copies concavity of the measurement in log–log coordinates.

### 4.3. A Comparison of Two Presented Ways

As documented above, an optimization of the nonlinear parameters of the models gives more realistic predictions if the LAOS data are preferentially taken into account. A consequent evaluation of steady shear viscosity does not have to be very precise but its functional course is more or less preserved. This contrasts to the situation when the parameters are based on steady shear viscosity measurements. In this case, a time-dependent course of normalized stress can be completely ignored as illustrated in Figure 9.

For both ways of parameters optimization, the values of the “anisotropic” parameter α in the Giesekus model attained values exceeding 0.5. However, as discussed in Bird et al. [38] and Schleiniger and Weinacht [39], the values of the parameter α exceeding 0.5 (α ∈ (0.5,1]) result in unphysical predictions as also documented in [27].

## 5. Conclusions

Predictive capabilities of four differential constitutive models (exponential PTT, Giesekus, Leonov, and modified XPP) were evaluated using 5 wt % solution of PEO in DMSO for which measured normalized stress exhibits a “protrusion” in otherwise smooth time course. It was shown that the optimization of the model’s nonlinear parameters should be based on the LAOS measurements rather than on the steady shear characteristics. The latter case results in predictions not corresponding to real responses when the studied material is deformed. If the LAOS-based model parameters are used, the modified XPP model provides a very good prediction of the steady shear viscosity. The exponential PTT model and the Giesekus model rather under-predicts and over-predicts the experimental data, respectively.

## Figures and Tables

**Table 1:** The dissipative term H(c) for the individual differential constitutive models.

Model	Dissipative Term H(c)
exponential Phan–Tien and Tanner (PTT) [30]	exp[ε (tr c−3)](c−I)
Giesekus [31]	$\alpha {\left(c-I\right)}^{2}+\;c-I$
Leonov [32]	$\frac{b}{2}\left(c^{2}-\frac{\mathrm{tr}\;c-\mathrm{tr}\;c^{-1}}{3}c-I\right)$
	$b=exp\left[-\zeta \left(\mathrm{tr}\;c-3\right)\right]+\frac{\sinh\left[\nu \left(\mathrm{tr}\;c-3\right)\right]}{\nu \left(\mathrm{tr}\;c-3\right)}-1$
modified extended Pom–Pom (mXPP) [33]	Fc−I
	$F=exp\left[\beta \left(\Lambda -1\right)\right]\left(2-\frac{1}{\sqrt{\Lambda }}\right)$, $\Lambda =\sqrt{\frac{tr\;c}{3}}$

**Table 2:** Relaxation spectrum and the nonlinear parameters of the constitutive models based on optimization of steady shear viscosity (SSV) and on fitting LAOS data (LAOS).

Linear Parameters					Nonlinear Parameters			
Maxwell Model			Exponential PTT Model		Giesekus Model	Leonov Model		Modified XPP Model
*i*	λ*_i_* [s]	*G_i_* [Pa]	ε*_i_* [-]	ξ*_i_* [-]	α*_i_* [-]	ζ*_i_* [-]	*ν_i_* [-]	β*_i_* [-]
			SSV/LAOS	SSV/LAOS	SSV/LAOS	SSV/LAOS	SSV/LAOS	SSV/LAOS
1	0.0055	502.1	4.3/3	0/0	0.8/0.9	0.1/0.8	6/0.55	8.1/8
2	0.0452	179.4	2.7/2	0/0	0.8/0.9	0.1/0.6	4.5/0.55	7.8/10
3	0.2045	75.59	1.3/0.28	0/0.09	0.7/1.0	0.1/0.4	3/0.55	7.2/2
4	0.9245	31.65	0.9/0.27	0/0.09	0.7/1.0	0.1/0.2	1/0.55	8.1/5
5	4.181	3.838	0.4/0.25	0/0	0.6/0.7	0.3/0.2	0.7/0.55	1.2/5
6	11.59	1.341	0.3/0.2	0/0	0.2/0.4	0.5/0.2	0.55/0.55	1.2/2
